# Communicating about the end of life: The path of prognostic awareness

**DOI:** 10.1017/S147895152400169X

**Published:** 2025-01-14

**Authors:** Jacopo D’Andria Ursoleo, Alice Bottussi, Andrew S. Epstein, Viviana Teresa Agosta, Fabrizio Monaco, William E. Rosa

**Affiliations:** 1 Department of Anesthesia and Intensive Care, IRCCS San Raffaele Scientific Institute, Milan, Italy; 2 Department of Medicine, Memorial Sloan Kettering Cancer Center, New York, NY, USA; 3 Department of Psychiatry and Behavioral Sciences, Memorial Sloan Kettering Cancer Center, New York, NY, USA

Patients with cancer are surviving longer, and therefore have more time both living as well as for end-of-life (EOL) planning (Bergenholtz et al. 2020). Major concerns for dying patients relate to issues such as dealing with pain and other distressing symptoms, as well as the loss of autonomy, agency, and functional independence (Steinhauser et al. 2000). Lack of control over the future is another common cause of anxiety and related suffering (Jackson and Emanuel 2024). Consequently, accurate prognostic understanding can enhance informed shared decision-making, improve quality of life and mood, and give patients the time and opportunity to make longer-term decisions for themselves, and arrangements for family members and caregivers (Jackson and Emanuel 2024). Unfortunately, fewer than 20% of cancer patients reports accurate prognostic awareness (PA) over the course of their illness, highlighting the need for a better focus on communicating prognostic information early and effectively (Loučka et al. 2021). Furthermore, due to the inherently challenging nature of these discussions, clinicians still find it difficult to engage in conversations about serious news, prognosis, goals of care, and dying and death (Hancock et al. 2007; Schenker 2024; Smith and Longo 2012). These hurdles can be more easily overcome by enhancing the skills and confidence of clinicians through effective communication strategies (Parry et al. 2014).

## The path to acceptance

In the face of advanced cancer, it is frequent for patients to oscillate between feelings of hopefulness, resignation, and realism (Avis et al. 2021; Kübler Ross 1969). At the EOL, hope and coping abilities are strongly interrelated: when facing the uncertain, holding hope fosters coping, and hope itself is, in a way, fostered by the underlying cognitive coping processes (Folkman 2010). Clinicians may believe that hope and the process of “personalizing the odds” (i.e., improving them in the light of personal or environmental attributes, existential beliefs, or on the basis of external information) could lead to patients’ being in a state of denial. Yet “unrealistic” hope (as deemed by clinicians) could still be considered an important part of the patient’s coping process (Folkman 2010). And, hope can take many forms – not just for a cure, but for other things, such as freedom from uncontrolled pain, more time, family time, and more. As such, clinicians should not only take advantage of patients’ hopes but also explore for what they hope, alongside showing empathy (Pollak et al. 2007) and slowly navigating the patient and their caregivers through the acknowledgement, recognition, and diversification of their hopes (Rosenberg et al. 2021). Carrying out iterative conversations over time allows the patient to achieve and sustain a deeper understanding of their condition, as well as to transition hope from unrealistic to realistic ones, which may be supported by newly acquired coping mechanisms (Folkman 2010; Jackson and Emanuel 2024). The clinicians’ aim is to guide the patient to reach the level of acceptance in adequate time for them to manage and prioritize the time they have left in a way that aligns with the patient’s values. Clinicians must also remember that for many patients, acceptance of dying may not truly occur early, consistently, or sometimes ever (Puri 2023).

### Prognostic awareness

Prognostic awareness (PA), defined as the capacity of the patient to develop both cognitive understanding of their condition and the ability to cope with it emotionally, may be one of the most important – albeit moving – targets to be addressed in the EOL setting (Jackson et al. 2024, 2013).

However, for multifaceted reasons (e.g., hope, anxiety, a belief in miracles, or even the nature of the PA questions themselves) (Derry et al. 2019b; Epstein et al. 2023; George et al. 2020a; Smith and Longo 2012), many patients with cancer exhibit an inaccurate comprehension of their prognosis and of the most likely trajectory of their illness (Epstein et al. 2016; Jackson et al. 2013). On the other hand, it is also well established that PA and – more generally – awareness of disease status are often achieved through participation in iterative discussions with health-care professionals (Epstein et al. 2016; Finlayson et al. 2024).

In this setting, the adoption of advanced communication techniques (Baile et al. 2000; Derry et al. 2019a; Epstein et al. 2021; Pollak et al. 2007; van Vliet and Epstein 2014) by clinicians should aim to progressively enhance the capacity of the patient to develop PA regarding their condition (Jackson et al. 2024, 2013). The earlier and more frequently that these clinician–patient conversations occur, the more likely a patient’s PA is to improve, as is a subsequent acknowledgment of prognosis and a reduction in anxiety from a feeling of lack of control (George et al. 2020b; Jackson et al. 2024).

The adoption of appropriate communication techniques (e.g., tailored, empathetic communication) is crucial to achieve an individualized and holistic EOL care while nurturing and fostering the development of PA (Finlayson et al. 2024). Though strategies such as advance care planning (ACP) have not been consistently shown to improve patient coping strategies (Bergenholtz et al. 2020; Korfage et al. 2020) recent studies of a patient/caregiver dyadic approach have shown promising results (Gray et al. 2021; Liu et al. 2024). Specialist input from palliative care may increase PA, and the early integration of palliative care interventions has been found to improve quality of life (Jackson et al. 2024), which may stem from palliative care’s holistic approach to all care domains (i.e., physical, psychological, spiritual, social) (Khayal and Barnato 2022).

Similarly, clinicians need to strike a balance between establishing and strengthening a patient’s understanding of their condition and their ability to cope and adapt to news of a poor prognosis (Back et al. 2003). Conversations should be framed to ask questions both about patients’ understanding of their illness and how they feel about it, such as: “*What do you know about your condition?*” with “*When you think about what lies ahead, what are you most worried about?*” (Jackson et al. 2024; van Vliet and Epstein 2014). Table 1A features 2 sets of questions with the goal of best supporting patients while trying to cultivate PA.

**Table 1.** (A) Communication strategies for cultivating prognostic awareness. (B) Best practices on how to communicate with a patient about terminal illness. CPR: cardiopulmonary resuscitation

**A**		
	**Prognostic awareness**	
**Discussion component**	**Illness/treatment understanding**	**Emotional coping**
**Initial prognosis**	*What do you understand about what I have just told you?* *This can be confusing. What would you like me to clarify?*	*What are you most worried about?* *What is the most challenging thing for you right now?*
**Prognostic uncertainty**	*Though people sometimes recover from this illness, I am worried that you might not.*	*If your health does not improve, what would be important to you?*
**Treatment alternatives**	*Let me outline the different possibilities of what could happen in the future…* *Given what I know about your priorities and your illness, I would recommend…*	*Do you think you would be able to cope with… or would you prefer…?* *Would this approach work for you?*
**Unrealistic hopes**	*It is good to be positive, but we can’t forget to look at all the possibilities.*	*I am glad to see you are so positive, and I too always hope for the best and will be here to help no matter what. As such, if your health were to decline, what would your priorities be?*
**Debunking false information**	*This is what we know to be the case…* *A number of my patients have also been asking about this…*	*I can see why you think this might be an option. The article/website seems convincing, but…*
**Checking physical comfort**	*Are you in much pain?* *Are you feeling comfortable?*	*Does your discomfort make you worry about how it will affect you and the people around you?*
**Checking progress**	*How is the treatment going for you?* *I’m sure you have some questions about your progress. What would you like to know?*	*Is the treatment what you hoped for?* *How are you dealing with the problems from the illness?* *Which part of the treatment is the most challenging for you?*
**Disclosure of terminal illness**	*I wish things were different, but unfortunately, the test results are not what we hoped they would be…* *The treatment has not worked in the way we had hoped…* *There are no further treatments that will help to control your cancer.* *I wish things were different, but, you are dying.*	*I am sorry that this is where things stand.* *Would you like me to let others know, and if so, do you want to be present when they hear?* *What can we do to make you more comfortable?*
**End-of-life decision-making**	*Would you like us to arrange for you to go to hospice, or would you like to go home* *May we discuss what life support (CPR and mechanical ventilation) means and if that is something you want to defer when the end of life comes? It can be helpful for patients and families to have these discussions early, so we know your wishes.*	*What do you think will be the most essential thing for you during this time?* *Some people see withholding CPR and mechanical ventilation as “giving up” but many embrace it as a decision that protects and liberates them from suffering and dependence on machines – what about you?*
**B**		
**Time**	Allow for adequate time to ensure that the conversation is not rushed.	
**Place**	Make sure you are somewhere private where you will not be overheard or interrupted.	
**Others present**	Ask whether they would like a family member, friend or caregiver to be present. In some cases, such as the patient having mild cognitive impairment, this is needed to make sure the patient understands and the information can be reinforced later. They may also require the emotional support.	
**Language**	Avoid confusing expressions such as “passing on” or “going to another place” and use concrete words such as “dying” and “death”. In an optimal situation where the patient has been clearly informed of possible outcomes at an early stage, the news will be less of a shock. If it is the first time death has been mentioned (e.g., due to an unexpected medical finding), build up to the disclosure rather than making a sudden declaration. Start by talking about the process that led to the discovery (see ‘Disclosure of terminal illness’ in Table 1A), but still avoid too much detail and medical jargon.	
**Check understanding**	Do not ask a yes/no question about whether the patient has understood. Ask, “*What is your understanding of what I have just told you?*”	
**Questions**	Do not simply ask, “*Do you have any questions?*” but ask more specifically, “*What do you think your family will need to know about this?*” or “*What do you think you need to do to prepare for this?*” Answer any resulting questions honestly and try to find out the answers to any questions you are unable to answer. The patient may not be in an emotional state to ask questions immediately, so let them know you will be available to ask questions at a later time. Be sure to respond to emotion, and elicit what patients and any loves ones are feeling after having discussed serious medical details.	
**Ending the conversation**	Do not leave the patient alone after the conversation. Make sure there will be someone with them to offer support. Ensure the patient and/family know how you or other clinicians can be reached, if needed, with more questions.	

### Building PA

Even when the clinician has clarified the nature of the cancer and limited treatment options available, patients may persist in overestimating their chances of survival. In such instances, it is imperative for the clinician to share in the hopes of the patient while actively supporting the patient to manage the important decisional milestones ahead. Clinicians should try to establish whether these beliefs are primarily due to a lack of clinical information or related to emotional or physical distress (Barnett 2006). Patients with terminal cancer are more likely to suffer from anxiety and depression if they were experiencing physical pain and were concerned about how these symptoms would affect their loss of autonomy and reliance on others (Barnett 2006). Consequently, clinicians need to take a patient’s physical discomfort into account alongside their psychological condition when assessing PA.

Depending on life expectancy, PA needs to be slowly developed to allow the patient time in coming to terms, as best as possible, with their condition. Rather than abruptly dispelling all unrealistic expectations with frank statements (e.g., “Miracles don’t happen”), the clinician needs to acknowledge and explore any such hopes with the patient empathically, allowing time for them to assimilate the information and reconcile it (Brenner et al. 2022). Over time, this ultimately enables patients to trust their clinicians, feel heard and understood, and express themselves emotionally, all which may help them develop a better understanding of their condition (Yanwei et al. 2017).

### The clinician’s approach

Patients often report a lack of clinician sensitivity and/or empathy in how they discuss EOL (Bernacki and Block 2014; Parker et al. 2007) while respect and empathy should be of the highest priority in all conversations (Jackson et al. 2024). Repeated and bidirectional conversations over time allow the patient and their caregivers to have the time needed to reframe their hopes and to acknowledge the terminal nature of their illness, while developing adequate coping skills and the ability to fully understand their condition (Jackson et al. 2024). In this scenario, ACP may prove beneficial by fostering the development of a trust-based therapeutic alliance and by supporting shared decision-making. Nevertheless, it is imperative for clinicians to note that this is – again – an iterative process, and one in which the patient’s expressed preferences may change along with both the course of the disease and the fluctuations in their understanding of their illness (Rosa et al. 2023a). In such conversations, the manifestation of strong emotions is to be expected and should be acknowledged, as opposed to met with factual explanations, justifications, or avoidant behaviors (Rosa et al. 2023b). The timing and place of clinician/patient conversations should also be considered according to the needs of individual patients. While some patients will report a lack of privacy where the conversations take place, others may not want to hear a poor diagnosis when alone and prefer to receive news in the presence of a caregiver (Bergenholtz et al. 2020). As such, an early part of the clinician/patient rapport should be documenting such information preferences and sharing them across involved care teams to enable more effective, person-centered later communication. This would involve asking questions such as: “*Are you comfortable talking here?*” or “*Would you like a family member present when we have conversations?*” from the first contact. Table 1B summarizes the main communicative aspects to be implemented by clinicians when informing a patient of the terminal nature of their illness.

As such, when approaching a clinical encounter with a patient affected by a life-limiting disease, the clinician should first consider and explore the patient’s cultural, spiritual, and social background and beliefs. By understanding the patient’s context, the clinician can better help the patient and respond to the patient’s and caregiver’s desire to discuss specific aspects of a serious illness. Each patient, at any moment in their life, may benefit from conversations focused on treatment and care options, developing a therapeutic alliance, or fostering connection and coping (Desai et al. 2018). Clinicians should ideally be able to adjust the course of the encounter and navigate these topics accordingly, ensuring a mutually beneficial and productive conversation. Adequate closure of the encounter is also crucial and requires providing space for questions, confirming mutual understanding, and planning future discussions to revisit important aspects of EOL care (Rosa et al. 2023b).

In conclusion, the goal of the clinician is to help the patient express their values and personhood while balancing their hopes with clinical realism. This process should be engendered with empathy and a seeking to understand who the patient is as a person, and what is most important to them so that clear goals and decisions can be established. Clinicians can create environments that also support patients to develop psychological and adaptive coping strategies as their PA changes over time. Timely disclosure of all possible outcomes, both positive and negative, constitutes an opportunity for patients to develop high levels of PA in the early stages of their illness, well before the EOL. Such a proactive approach enables patients to cope emotionally, make decisions, and address practical considerations over an extended timeframe.

## Data Availability

Further information is available from the corresponding authors upon reasonable request.
